# Supplementary material for: Tobacco Plant Growth-Promoting and Antifungal Activities of Three Endophytic Yeast Strains
Source: Plants (Basel). 2022 Mar 11;11(6):751. doi: 10.3390/plants11060751 (PMC8953121; doi:10.3390/plants11060751)
Supplement: Supplementary file 1 [file plants-11-00751-s001.zip › plants-1588405-supplementary.pdf]

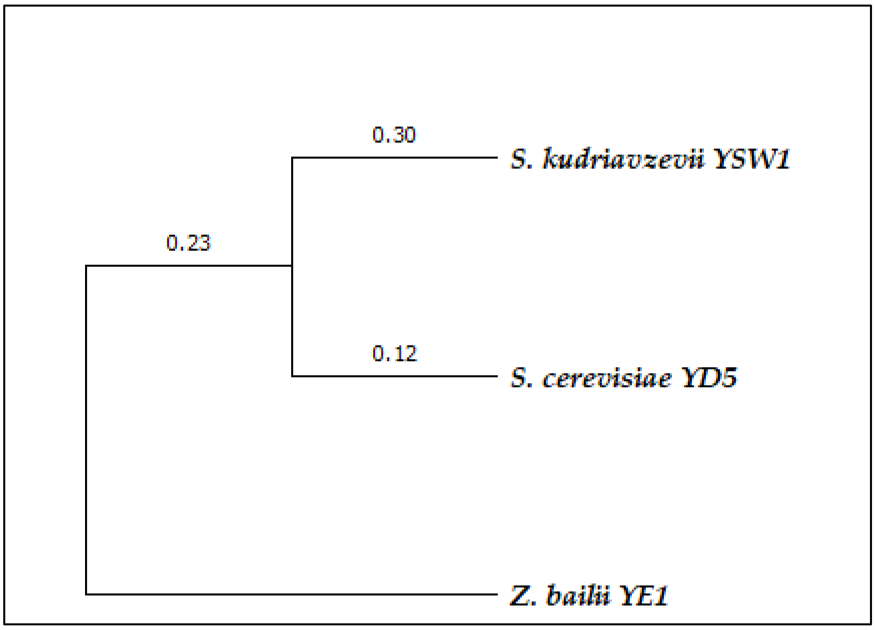

Supplimentary Figure S1. A dentrograme was inferred using the Neighbor-Joining method .. This analysis involved 3 nucleotide sequences. There were a total of 1199 positions in the final dataset. Evolutionary analyses were conducted in MEGA X softwaer [Kumar S., Stecher G., Li M., Knyaz C., and Tamura K. (**2018**). MEGA X: Molecular Evolutionary Genetics Analysis across computing platforms. *Molecular Biology and Evolution* **35**:1547-1549].

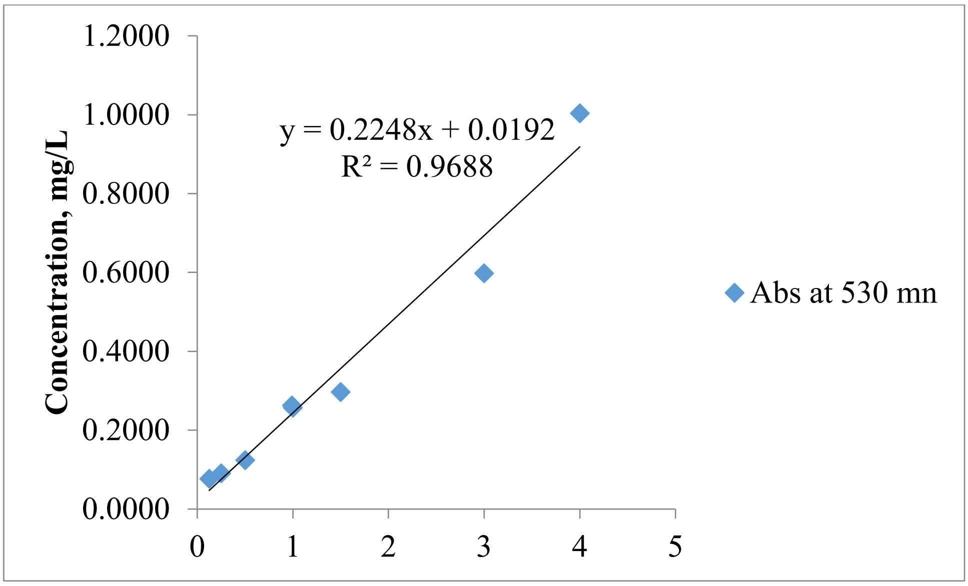

Supplimentary Figure S2. *Standard curve* of *IAA production by yeast strains.*
